# B7-H4 promotes tumor growth and metastatic progression in lung cancer by impacting cell proliferation and survival

**DOI:** 10.18632/oncotarget.14475

**Published:** 2017-01-03

**Authors:** Xiuqin Zhang, Liming Cai, Guangbo Zhang, Yu Shen, Jianan Huang

**Affiliations:** ^1^ Department of Respiratory Medicine, The First Affiliated Hospital of Soochow University, Suzhou 215006, China; ^2^ Clinical Immunology Laboratory of Jiangsu Province, Suzhou 215006, China; ^3^ Department of Respiratory Medicine, The Affiliated Hospital of Jiangnan University (WuXi No.4 People's Hospital), Wuxi 214000, China

**Keywords:** B7-H4, lung cancer, tumorigenesis, metastasis

## Abstract

Aberrant expression of B7-H4 occurs across a broad spectrum of human cancers. The aim of this study was to investigate the key role of B7-H4 during tumorigenesis and metastasis of human lung cancer. Our data showed that the shRNA-mediated disruption of B7-H4 markedly inhibited tumor cell proliferation, invasion and migration, increased cell apoptosis and arrested cell cycle at G0/G1. These changes were accompanied by a marked increase in Bax and caspase-3/caspase-8, but a decrease in Bcl-2, cyclinD1 and activation of AKT. In addition, our shRNA-mediated disruption of B7-H4 led to a marked decrease in tumor growth in the immune-compromised mice. Importantly, B7-H4 was expressed in 53.33% of lung carcinomas from our patient cohort (*n* = 90), but not in any of adjacent non-cancerous tissues, according to our IHC analyses. In particular, B7-H4 expression appeared to be associated with lymph node metastasis (*P* = 0.008) and TNM stage (*P* = 0.012). Taken together, our study demonstrates a strong promoting role of B7-H4 in lung tumor growth, progression and metastasis, and supports its potential as a therapeutic target for the treatment of the disease.

## INTRODUCTION

Lung cancer is one of the leading causes of cancer-related death in men and the second cause (after breast cancer) in women worldwide [1]. Alarmingly, more than 40% of patients being initially diagnosed with lung cancer have acquired the advanced-stage disease [2]. While many standard therapies of lung cancer are available, including surgery, chemotherapy, radiotherapy as well as targeted and combined therapies, the overall survival of lung cancer patients remains poor [3]. Therefore, there is an urgent need for development of better predictive biomarkers and target-based therapies for improving the diagnosis and treatment of this aggressive disease.

B7-H4, which is also known as VTCN1, B7x or B7S1, is an important member of the B7 family. It has been shown to control cytokine secretion, cytotoxicity development and activation of T cells [4–6]. B7-H4 is also implicated as a promising biomarker and a candidate therapeutic target for multiple types of human cancers [7–13]. Notably, the study by Safaei et al. demonstrates that B7-H4 is a strong prognostic value and an independent predictor marker for human renal cell carcinoma [7]. There is also evidence that B7-H4 expression correlates with patients’ survival and tumor immune response in human melanoma [9]. A similar observation is reported for the patients with esophageal squamous carcinomas [11]. Together, these studies suggest that B7-H4 is a crucial driver of development and progression of human carcinomas. In contrast, there is little known about its role in lung cancer, even though being strongly implicated in the regulation of tumor immunity.

Here, we applied shRNA-mediated gene silencing technology to investigate the functional and signaling roles of B7-H4 in lung tumor growth and progression, as well as underlying molecular mechanisms. Results from our *vitro* and *vivo* analyses demonstrate that B7-H4 is a key driver and a promising therapeutic target for human lung cancer.

## RESULTS

### Altered expression of B7-H4 in human tumor and non-tumor specimens

**Figure 1:**
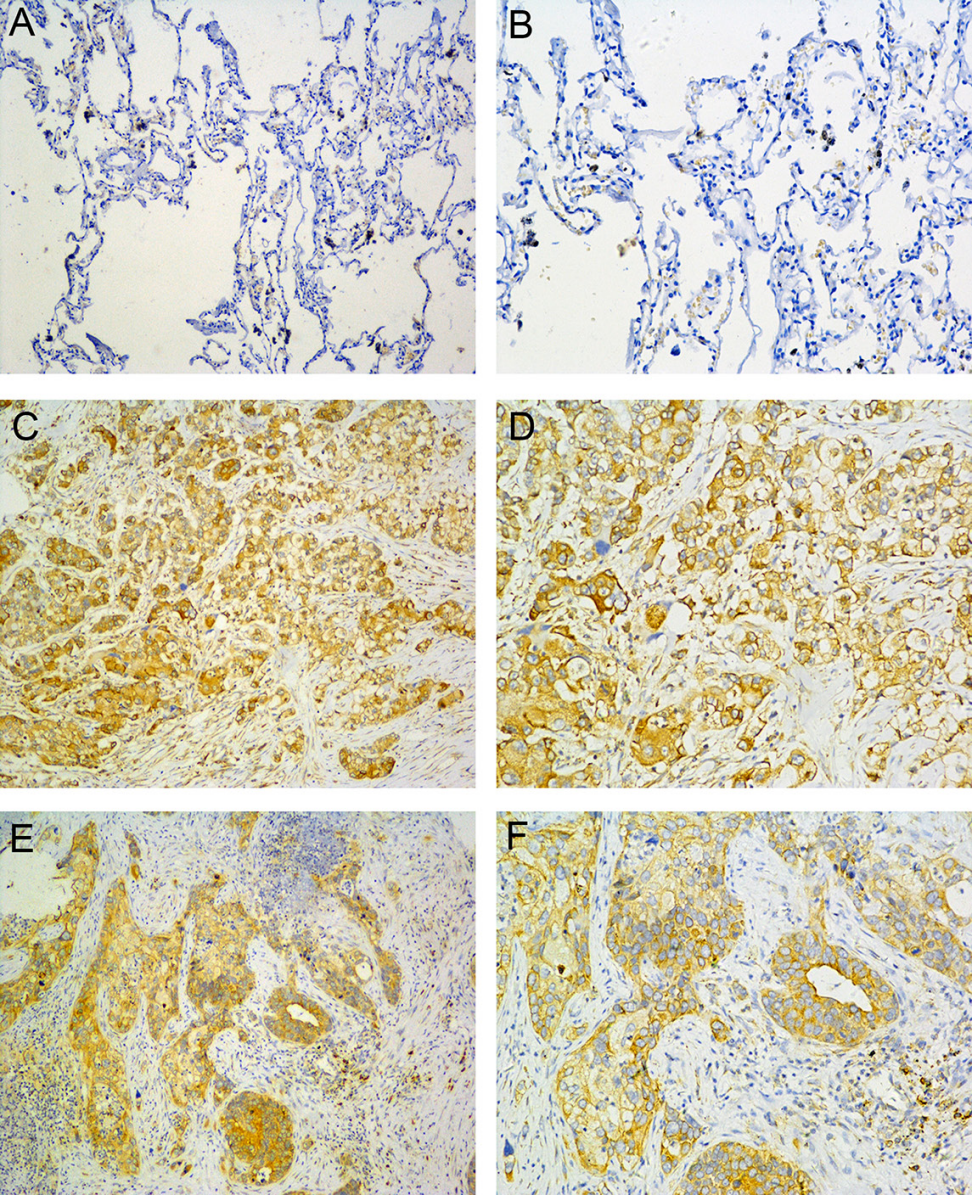
B7-H4 expression in NSCLC (**A**, **B**) normal lung tissue; (**C**, **D**) Adenocarcinoma; (**E**, **F**) Squamous cell carcinoma. (left: SP ×100, right: SP ×200).

To evaluate the clinical importance of B7-H4 in lung cancer, we conducted IHC analyses of paraffin–embedded tumors and adjacent non-tumor tissues of lung cancer patients. As shown in Figure 1, B7-H4 protein expression was detected in 48/90 (53.33%) lung carcinomas and 0/50 (0%) non-tumor tissues. In addition, B7-H4 expression was found in the cytoplasm and membrane of cancer cells. These data indicate that the B7-H4 expression is significantly upregulated in lung tumors in our current patient cohort, compared to their adjacent non-cancerous tissues.

### Association between B7-H4 expression and clinicopathological characteristics

Next, we assessed the link between B7-H4 expression and key clinicopathological parameters for our lung cancer patient cohort. As shown in Table 1, B7-H4 expression was associated with lymph node metastasis and pathologic stage (respectively *p* = 0.008, *p* = 0.012). However, there was lack of association between B7-H4 expression and patients’ age, gender, smoking status, pathological type, differentiation and three year survival rate after surgery. Thus, our data imply a strong role of B7-H4 in lung cancer progress and lymph node-associated metastasis.

**Table 1:** Correlation of B7-H4 expression with clinicopathologic parameters of NSCLC

Clinical characteristics	Case number	B7-H4 expression		*P* value
		Positive	Negative	
**All cases**	90	48	42	
**Age (years)**				
< 60	42	20	22	0.309
≥ 60	48	28	20	
**Gender**				
Male	59	35	24	0.116
Female	31	13	18	
**Smoking status**				
Smoker	55	30	25	0.773
Non-smoker	35	18	17	
**Pathological type**				
Adenocarcinoma	60	29	31	0.179
Squamous cell carcinoma	30	19	11	
**Differentiation**				
Well	25	15	10	0.432
Not well	65	33	32	
**Pathologic lymph node**				
N0	34	12	22	0.008^*^
N1+N2+N3	56	36	20	
**Pathologic stage**				
I	25	8	17	0.012*
II+III	65	40	25	
**Survival years after surgery**				
< 3 year	66	38	28	0.181
≥ 3year	24	10	14	

Note: *P* values were calculated using chi-square test; *p < 0.05, ^*^p < 0.01.

### Lentivirus-mediated expression of B7-H4-specific shRNA in A549 cells

**Figure 2:**
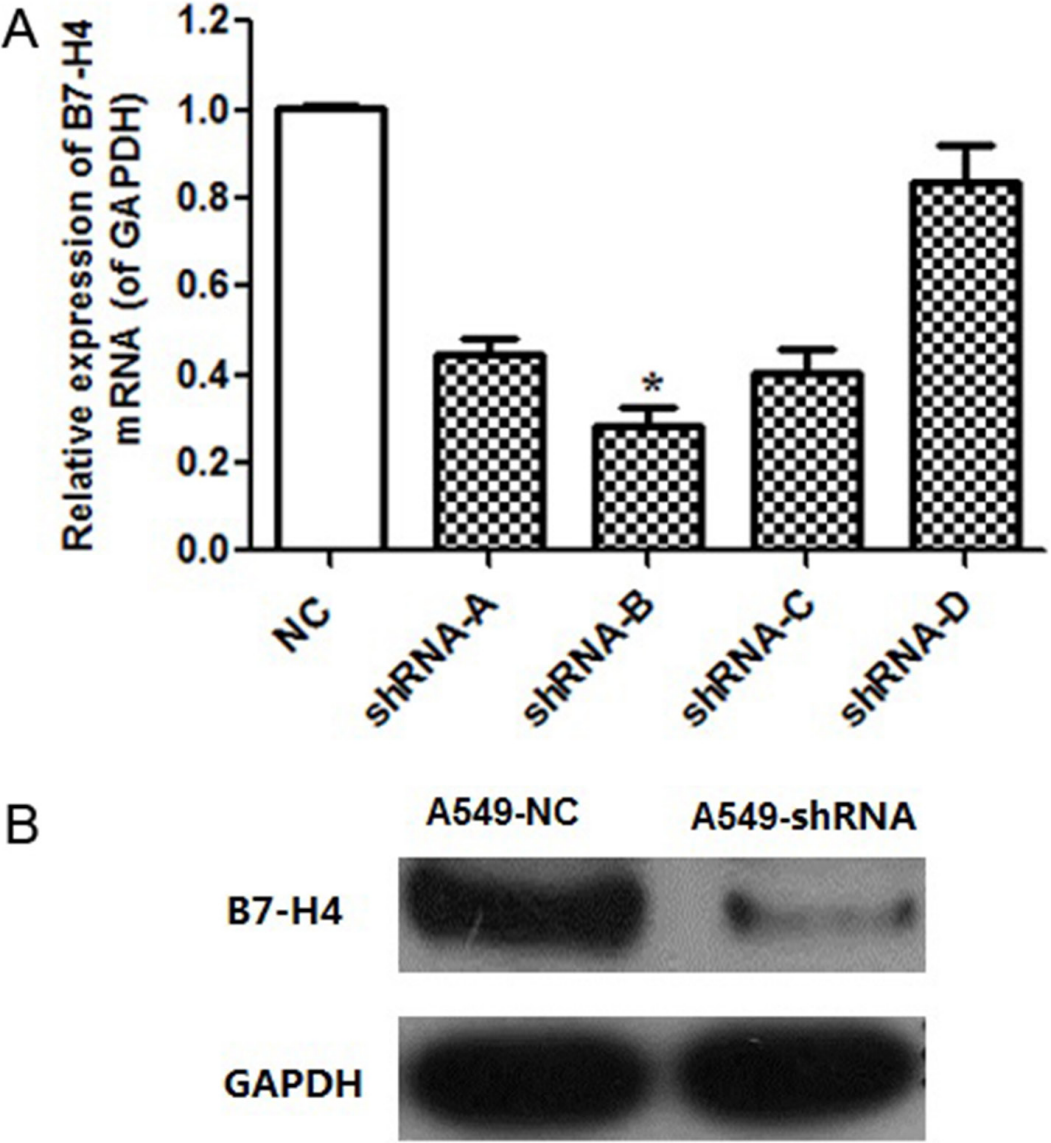
B7-H4 knockdown effect in mRNA and protein level (**A**) B7-H4 mRNA level was determined by quantitative RT-PCR. The level of B7-H4 mRNA expression in A549-shRNA-B cells was decreased by approximately 71.9% compared with A549-shRNA-A, A549-shRNA-C or A549-shRNA-D. (**B**) The level of B7-H4 protein expression in A549-shRNA-B cells was decreased significantly compared with A549-NC cells by western blot. Data was presented as the mean ± SD; *p < 0.05.

Human lung cancer cell line A549, which expresses high level of endogenous B7-H4, was applied for our subsequent functional analyses. Here, four different B7-H4 shRNAs were designed to silencing the expression level of B7-H4 gene in A549 cells. With non-specific control shRNA (negative control, NC), B7-H4 mRNA expression level was evaluated by real-time PCR. As shown in Figure 2A, B7-H4-shRNA in A549-B group provided the highest silencing efficiency significantly compared with A549-NC and its knockdown efficiency is up to 71.9% (*p* < 0.05). Meanwhile, the B7-H4 protein expression level in A549-B was lower than A549-NC group (Figure 2). Therefore, the A549-shRNA-B stable line, named as A549-shRNA cells, was used for our subsequent analyses.

### A role of B7-H4 in tumor cell proliferation

**Figure 3:**
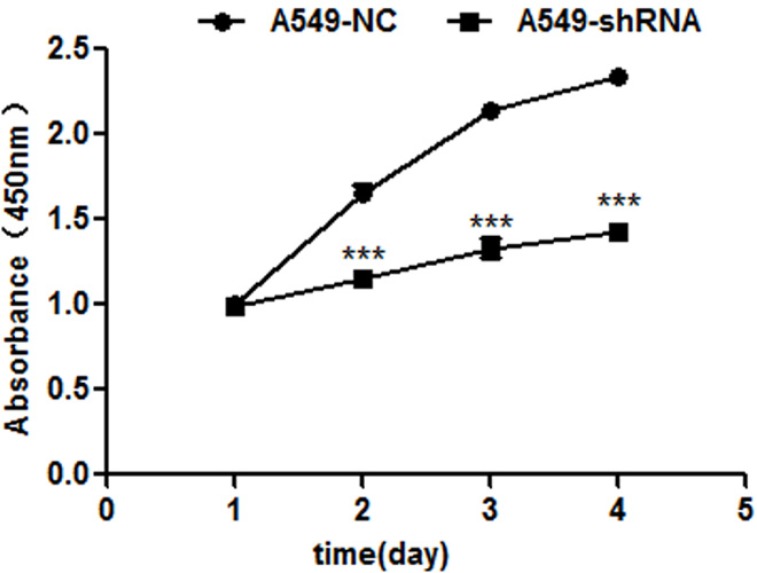
B7-H4 shRNA inhibits cell proliferation by CCK-8 assay 1–4 days The viabilities of A549-shRNA cells and A549-NC cells were detected at 450 nm. Data was presented as the mean ± SD; ***p < 0.001.

The potential effect of RNAi-mediated B7-H4 down-regulation on A549 cell growth was evaluated by CCK-8 assay. In Figure 3, the results showed that A549-shRNA cell growth rate was significantly reduced on day 4, as compared with A549-NC cells (*p* < 0.001).

### A link of B7-H4 to tumor cell migration and invasion

**Figure 4:**
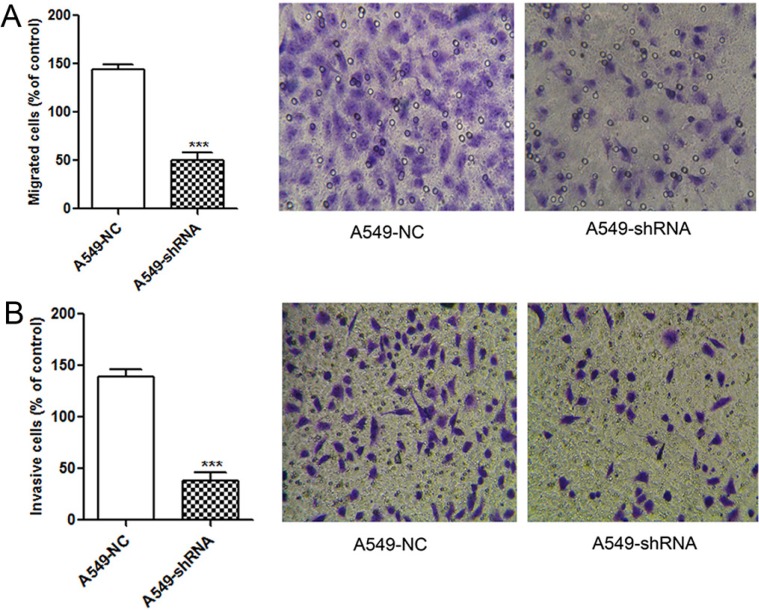
Reducing migration and invasion abilities of A549 cells by lentivirus-mediated RNAi (**A**) Effect on tumor migration activity of A549-shRNA cells. A549-shRNA cells and A549-NC cells that migrated through the upper chamber with 8-μm pores membrane were counted and photographed under a light microscope, respectively (SP ×200). (**B**) Effect on tumor invasion activity of A549-shRNA cells. A549-shRNA cells and A549-NC cells that invaded through the Matrigel coated inserts were counted and photographed under a light microscope, respectively (SP ×200). Data was presented as the mean ± SD; ***p < 0.001.

To address the role of B7-H4-shRNA in the aggressive behaviors of A549 cells, we evaluated the influence of B7-H4-shRNA on migration and invasion with transwell assays. As shown in Figure 4, abilities of migration and invasion were significantly inhibited in A549-shRNA cells obviously, compared to that in A549-NC cells (*p* < 0.001). These data demonstrate a promoting role of B7-H4 in the motility and promoted invasion of A549 cells.

### A role of B7-H4 in cell cycle progression and apoptosis

**Figure 5:**
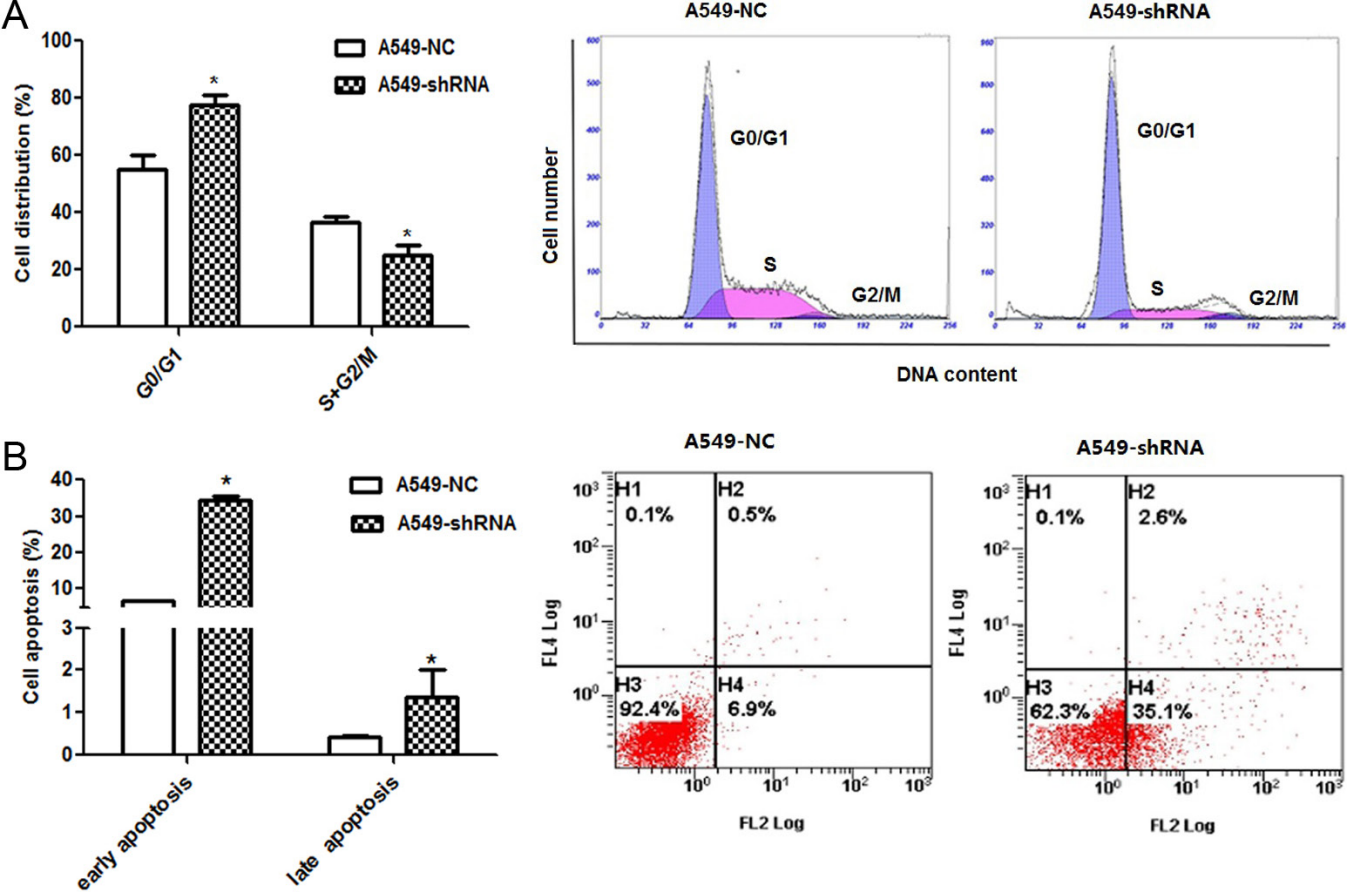
Effect of B7-H4 shRNA on cell circle distribution and apoptosis in A549 cells (**A**) Cell cycle distribution was identified by flow cytometry. A549-shRNA cells were arrested in the G0/G1 stage of cell cycle significantly compared with A549-NC cells; (**B**) Cell apoptosis of A549 cells was detected by by flow cytometry. Data was presented as the mean ± SD; *p < 0.05.

Moreover, the influence of B7-H4 gene silencing on tumor cell cycle was assayed. In Figure 5A, the flow cytometry analysis showed that A549-shRNA cells were arrested in the G0/G1 stage of cell cycle compared to A549-NC cells (77.70 ± 6.26% and 55.25 ± 8.61%, *p* < 0.05); while the percentage of S+G2/M phase in A549-shRNA cells were decreased relative to A549-NC cells (24.96 ± 6.10% and 36.66 ± 2.96%, *p* < 0.05). The results showed that the percentage of G0/G1 was increased and the proportion of S+G2/M phase was decreased upon B7-H4 ablation. The data indicated that inhibition of B7-H4 resulted in G0/G1 arrest. The G0/G1 arrest in cell cycle could inhibit cell growth and proliferation of cancer. At same time, we detected cell apoptosis in above cells by using Annexin V–PE and 7-ADD double staining. In Figure 5B, the results showed that the percentage of early apoptosis cells in A549-shRNA was higher than the A549-NC (34.53 ± 1.44% and 6.87 ± 0.15% *p* < 0.05); there was no difference in percentage of late apoptosis between the two groups (1.37 ± 1.10% and 0.43 ± 0.06%, *p* > 0.05). It indicated that inhibition of B7-H4 expression could promote apoptosis of tumor. These results suggested that B7-H4 contributed to tumor progression by apoptosis inhibition.

**Figure 6:**
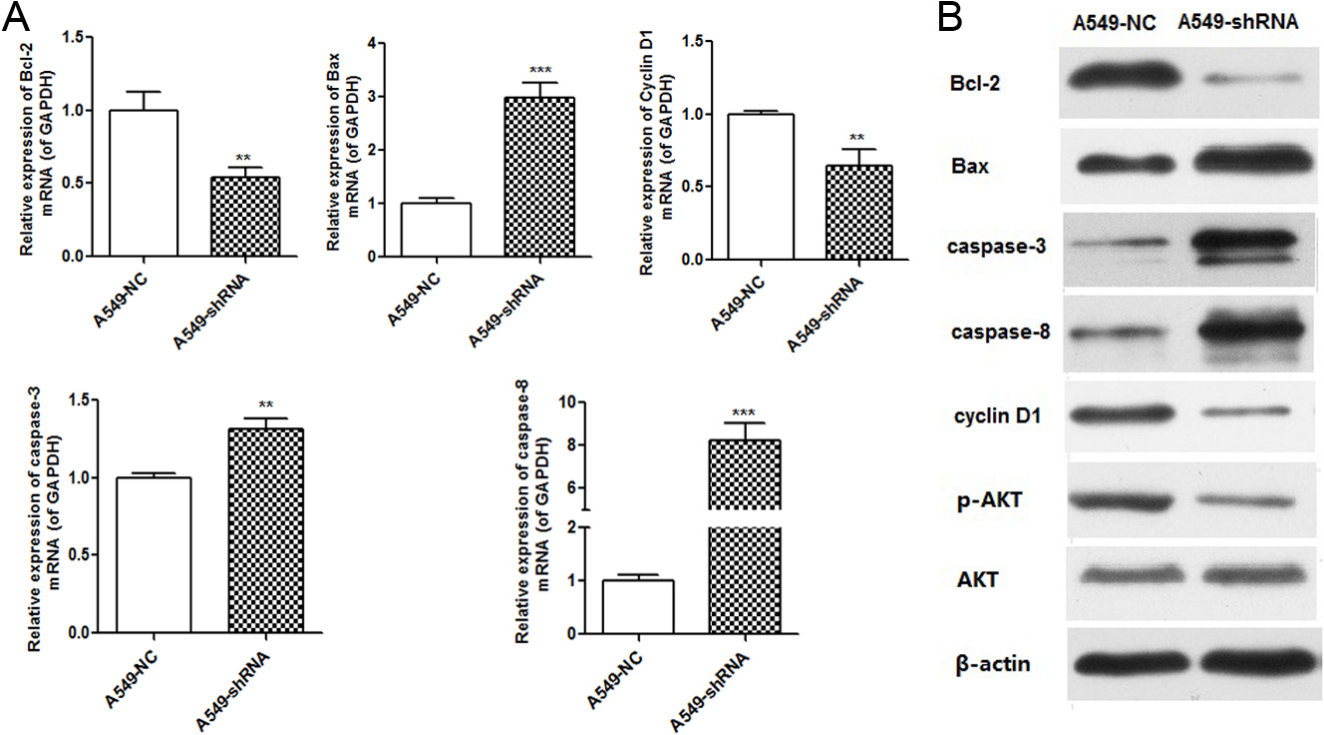
B7-H4 RNAi influenced expression of molecules associated with cell cycle and apoptosis (**A**) Quantification of mRNA expression of molecules associated with cell cycle and apoptosis. (**B**) Protein expression of molecules associated with cell cycle and apoptosis. Data was presented as the mean ± SD; **p < 0.01, ***p < 0.001.

To investigate which apoptosis and cell cycle pathway components were involved in A549 cells response to B7-H4 RNAi, we detected the expression of apoptosis and cell cycle associated molecules by real time PCR and Western blot. We observed the increase of the pro-apoptotic Bax, decrease of anti-apoptotic Bcl-2 and cell cycle associated cyclinD1, and activation of caspase-3 and caspase-8 relative to controls. Furthermore, knockdown of B7-H4 decreased AKT phosphorylation, but not changed total AKT protein expression level in A549-shRNA cells (Figure 6).

### Effect of B7-H4 knockdown on tumor growth *in vivo*

**Figure 7:**
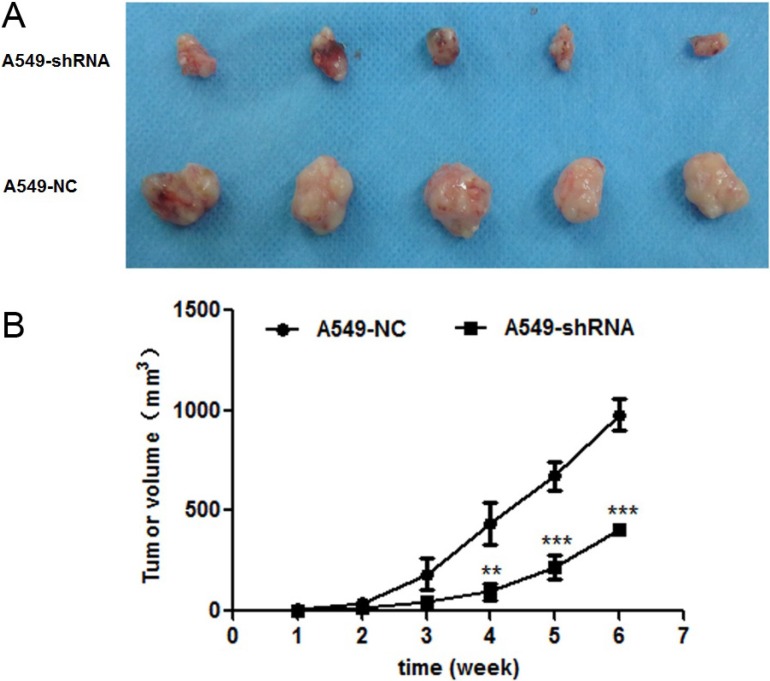
Effect of B7-H4shRNA on lung cancer *in vivo* (**A**) Images of xenografted tumors developed from A549 cells with B7-H4 shRNA and control. (**B**) Plot of changes in tumor volumes (mm^3^) over time. Size of tumors were measured over a period of 6 weeks with conrol group. Data was presented as the mean ± SD; **p < 0.01, ***p < 0.001.

To confirm the function of B7-H4 in lung cancer *in vivo*, the xenograft tumor model of BALB/c nude mice was established by injecting stable A549-shRNA cells and A549-NC cells into the groin of nude. The sizes of tumor volume were shown in Figure 7. The results demonstrated that the tumor volume sizes of nude mice injected with A549-shRNA cells were smaller than nude mice injected with A549-NC cells (*p* < 0.001). Our data indicated that B7-H4 gene silencing results in inhibition of tumor growth.

## DISCUSSION

To date, B7-H4 gene has been intensively studied in many tumors [8, 14–18]. Here, our study provides key evidence on the role of B7-H4 in tumorigenesis and metastasis of human lung cancer. In particular, the B7-H4 expression is significantly elevated in human lung tumor tissues, while being absent in normal tissues. More importantly, the relationship between B7-H4 expression and clinicopathological factors shows, B7-H4 expression is strongly correlated with lymph node metastasis and pathologic stage.

Our *in vitro* and vivo studies have demonstrated a strong role of B7-H4 in tumor growth and metastatic progression of lung cancer by use of the RNA interference approach. Notably, our data showed that B7-H4 gene silencing markedly inhibited cell proliferation, invasion and migration, increased cell apoptosis and arrested cell cycle at G0/G1. Apoptosis is critical step for tumor development. The induction of apoptosis is a significant therapeutic strategy for tumor [19]. Bcl-2 blocks apoptosis by preventing pro-apoptotic molecules from mitochondria into the cytosol, while Bax promotes apoptosis by inducing mitochondrial outer membrane permeabilization [20–23]. Increased levels of pro-apoptotic proteins and/or decreased anti-apoptotic proteins can lead to apoptosis. In this study, A549 cell transfection with B7-H4-shRNA led to decrease of anti-apoptotic protein Bcl-2 and increase of the pro-apoptotic protein Bax. As we known, it is the Bax/Bcl-2 ratio that controls the ultimate sensitivity to cell death stimuli [24]. This alteration was responsible for the caspase-8 and caspase-3 activation and the induction of apoptosis. Cell cycle progression is also a key step for tumor growth. Targeting cell cycle is also an obvious therapeutic strategy for human cancer [25]. It is well established that the abnormal cell cycle happens in almost all cancers, which is an unavoidable procedure in carcinoma progression. In this article, B7-H4 gene silencing blocked the transformation from G0/G1 phase to S and G2/M phase, and followed decrease of cyclin D1 expression. There are complex links between cell cycle and cell apoptosis in tumor. Generally, the apoptosis of cells follows with cell cycle arrest [26]. B7-H4 might be closely bounded with the apoptosis of A549 cells through inhibition of cell cycle.

It is worth noting that the orthotopic xenograft tumor model of nude mice was also established to confirm the effect of B7-H4 on the growth of tumors *in vivo*. Two groups of xenograft growth provide direct evidences that B7-H4 gene RNAi resulted in the obvious variation in tumor mass. It indicated that B7-H4 gene silencing suppress growth of xenografts *in vivo* and has a potent therapeutic effect against tumor cells.

The aberrant activation of AKT signaling pathway has a critical influence in many biological processes of human malignancies, including NSCLC [27]. Knockdown of AKT expression suppressed lung cancer cell proliferation [28], and the molecules correlated with apoptosis such as Bcl-2 and caspase-3 are the downstream targets of Akt [29]. In this study, B7-H4 gene silencing induced by B7-H4 shRNA inhibited AKT phosphorylation. AKT phosphorylation inhibition by B7-H4 gene silencing suggested that B7-H4 inhibition could be responsible for decreasing tumor cell oncogenicity, increasing apoptosis, and arresting cell cycle procedure through AKT pathway. It indicates that B7-H4 and AKT signaling pathway might have a bound relationship, Liang Zhang et al. reported that B7-H4 was a cytoplasmic-nuclear shuttling protein, and B7-H4 promoted tumor progression and cell proliferation through translocating into nucleus [30]. But the link between B7-H4 and AKT signaling pathway needs to be furtherly investigated.

In summary, our work from vivo and vitro demonstrated that B7-H4 might be an important cancer promoter and a novel therapeutic target for human lung cancer. B7-H4 inhibition might offer an exciting opportunity to inhibit the progression and metastasis of human lung cancers.

## MATERIALS AND METHODS

### Cancer tissues specimens

Formalin-fixed, paraffin-embedded tumor tissues were obtained from 90 cases of lung carcinomas and 50 cases of adjacent non-tumor specimens (within at least 5cm of tumor margin) were also obtained in Affiliated Hospital of Jiangnan University (WuXi No.4 People's Hospital is renamed as the Affiliated Hospital of Jiangnan University), China. The protocol in this study was approved by the ethics committees of the Affiliated Hospital of Jiangnan University.

### Immunohistochemical staining

The paraffin-embedded human lung tumor tissues were cut into 4μm-thick serial sections, Immunohistochemical (IHC) staining was performed on human tumor specimens and non-tumor specimens by using Envision methods, these slides were labeled by using primary antibodies specific for B7-H4 (GeneTex, Merck & Millipore), a negative control was carried out by replacing the primary antibody with PBS, then sections were incubated with horseradish peroxide-labeled goat anti-Rabbit second antibody (Merck & Millipore). Semi-quantitative measurements of staining intensity (0–3, i.e., least intense to most intense) and the proportion of stained cells (0–4, i.e., no cells stained to more than 70% cells stained) were determined as previously described [31]. All slides were examined and evaluated independently by two pathologists.

### Cells culture

A549 cells, derived from human lung adenocarcinoma, were purchased from cell bank of Chinese Academy of Science, Shanghai, in China, and maintained in Roswell Park Memorial Institute (RPMI, Gbico) medium containing 10% fetal bovine serum (Gbico), 1% penicillin and streptomyc (Life Technologies) at 37°C, in a humidified incubator with 5% CO2.

### Cloning and expression of B7-H4 shRNA

Four DNA template oligonucleotides corresponding to B7-H4 gene were designed and synthesized. Four pairs of B7-H4 specific shRNA were also synthesized according to four DNA template oligonucleotides sequence. The synthesized single DNA chain was ligated with empty vector GV115 (hU6-MCS-CMV-EGFP, Shanghai genechem Co., Ltd. China). The connected product was transferred to Escherichia coli and cultured on the Amp+LB plate for an over night at 37°C. The positive colonies were selected and amplified using PCR primers, then inoculated into Amp+LB liquid medium in shaking bottle at 37°C for an over night. The plasmid was extracted by using Axygen plasmid extraction kit (Qiagen, German) and sequenced (invitrogen). Recombinant lentiviral vector was then transfected into 293T cells. Supernatants containing lentiviruses were harvested 48h later after transfection. A549 cells were infected with viral supernatants for 12h at an appropriate concentration of viral supernatants. After 3 days post-transfection, 90% of the cells were transfected by observing the expression of GFP. There were respective two experimental groups for A549 cells: cells transfected with lentivirus mediated shRNA-targeted B7-H4 were named A549-shRNA-A, A549-shRNA-B, A549-shRNA-C, A549-shRNA-D cells, cells transfected with lentivirus mediated shRNA (negative control, NC) were named A549-NC cells.

### Real-time reverse transcription-PCR

A549-shRNA cells and A549-NC cells were seeded and harvested after culture 96h. Total RNA was extracted from each group of cells using the Trizol (Invitrogen), cDNA synthesis was obtained using the ThermoScript^TM^ Reverse Transcriptase kit (Life Technologies), then, RT-PCR was performed using Power SYBR^®^ Green PCR Master Mix (Life Technologies). The mRNA expression of various genes was assessed using ABI 7700 Sequence Detection System (Applied Biosystems). Relative gene expression was quantified.

### Cell proliferation assay

The cell viability of A549-shRNA and A549-NC cells were measured by Cell Counting Kit-8Assay (Dojindo, Japan). Two groups of cells, at 1 × 10^5^/well, were respectively seeded into 96-well plates, and incubated in the water-saturated carbon dioxide incubator. The absorbance at 450 nm was measured by using a Model AMR-100 microplate reader.

### Cell apoptosis and cycle assays

Apoptosis assays were performed by using an Annexin V–PE apoptosis detection kit (BD PharMingen). A549-shRNA and A549-NC cells in 6-well plate were digested and collected at 96h, cells were suspended in PBS and stained with Annexin V–PE and 7-ADD (Dojindo, Japan), and detected in a FACS Calibur cytometer (FACS Caliber, BD Biosciences). Annexin V-PE and 7-ADD double-negative cells were considered to be non-apoptotic cells. PI staining kit (Vazyme) was used to detect cycle of cells by using flow cytometry. DNA content of cell was detected according to the relative proportions of cells in the G1/G0, S and G2/M phases of the cell cycle.

### Cell migration and invasion assays *in vitro*

In migration assay, A549-shRNA and A549-NC cells (1 × 10^5^ cells respectively) suspended with serum-free RPMI medium were seeded to upper chamber with 8 μm pores membrane, and 10% FBS contained medium were added into lower chamber, then cells were incubated for 12h at 37°C. Cells removed from upper chamber to lower chamber. In invasion assay, A549-shRNA and A549-NC cells ( 2.0 × 10^4^ cells respectively) suspended with serum-free RPMI medium were seeded to upper chamber, 10% FBS contained medium were placed in the lower chamber. Then cells were incubated for 24 h at 37°C. Cells were removed from upper chamber surface of 8 μm pores polycarbonate filter coated with 1mg/ml Matrigel (Corning, Inc.) to lower chamber. The cells on the lower surface of the filter were fixed in methanol and stained with 0.1% crystal violet. The stained cells were washed three times with PBS, and counted in five random high-power fields.

### Tumor growth *in vivo*

Briefly, A549-shRNA cells and A549-NC cells (both 2.0 × 10^7^ cells) were injected into groin of 8-week-old male BALB/c nude mice. The size of tumors was observed at intervals of 3 days. The orthotopic xenograft tumors were measured using a caliber and harvested at 42 days after the injection.

### Western blot

A549-shRNA cells and A549-NC cells were lysed in 50ul lysis buffer (Beyotime) on ice for 30 min respectively. Cells lysates were centrifuged and denatured with SDS polyacrylamide gel electrophoresis (SDS-PAGE) sample buffer for 10 min at 95°C. Proteins were electrophoresed on 10% SDS-PAGE gels and transferred to polyvinylidene difluoride (PVDF) membranes. The membranes were blocked with 5% skim milk in TBST and then incubated with specific antibodies (including B7-H4, GeneTex, Merck & Millipore; Bcl-2/Bax, caspase-3/caspase-8, p-AKT/AKT, CyclinD1 and GAPDH, Cell Signaling Technology, Inc.) for 2 h at room temperature. After thorough washes, membranes were incubated with horseradish peroxidase (HRP) conjugated secondary antibody (Santa Cruz, CA).

### Statistical analysis

The association between B7-H4 expression and clinic pathologic features was analyzed by chi-square test. The difference between the groups showed mean ± standard deviation (*x* ± SD), the differences between the groups was analyzed by two-tailed Student's *t*-test. The software of SPSS version17.0 (SPSS, Inc.) was used for statistical analysis. Statistical significance was considered at *p* < 0.05 and markedly significance was considered at *p* < 0.01.
